# Tolerance levels of mass density for adaptive helical tomotherapy using MVCT

**DOI:** 10.1093/jrr/rrac071

**Published:** 2022-11-14

**Authors:** Shogo Tsunemine, Shuichi Ozawa, Minoru Nakao, Hideharu Miura, Akito Saito, Daisuke Kawahara, Yasuhiko Onishi, Takashi Onishi, Fumito Okawa, Atsushi Terai, Taiki Hashiguchi, Hidetoshi Yamasaki, Tsutomu Maruta, Yuji Murakami, Yasushi Nagata

**Affiliations:** Program of Medicine Doctoral Course, Graduate School of Biomedical and Health Sciences, Hiroshima University, 1-2-3, Kasumi, Minamiku, Hiroshima, 734-8553 Japan; Department of Radiology, National Hospital Organization Himeji Medical Center, 68, Hommachi, Himeji, Hyogo, 670-8520, Japan; Department of Radiology, National Hospital Organization Himeji Medical Center, 68, Hommachi, Himeji, Hyogo, 670-8520, Japan; Hiroshima High-Precision Radiotherapy Cancer Center, Hiroshima, 3-2-2, Futabanosato, Higashiku, Hiroshima, 732-0057, Japan; Department of Radiation Oncology, Graduate School of Biomedical and Health Sciences, Hiroshima University, 1-2-3, Kasumi, Minamiku, Hiroshima, 734-8553 Japan; Hiroshima High-Precision Radiotherapy Cancer Center, Hiroshima, 3-2-2, Futabanosato, Higashiku, Hiroshima, 732-0057, Japan; Department of Radiation Oncology, Graduate School of Biomedical and Health Sciences, Hiroshima University, 1-2-3, Kasumi, Minamiku, Hiroshima, 734-8553 Japan; Hiroshima High-Precision Radiotherapy Cancer Center, Hiroshima, 3-2-2, Futabanosato, Higashiku, Hiroshima, 732-0057, Japan; Department of Radiation Oncology, Graduate School of Biomedical and Health Sciences, Hiroshima University, 1-2-3, Kasumi, Minamiku, Hiroshima, 734-8553 Japan; Department of Radiation Oncology, Graduate School of Biomedical and Health Sciences, Hiroshima University, 1-2-3, Kasumi, Minamiku, Hiroshima, 734-8553 Japan; Department of Radiation Oncology, Graduate School of Biomedical and Health Sciences, Hiroshima University, 1-2-3, Kasumi, Minamiku, Hiroshima, 734-8553 Japan; Department of Radiology, National Hospital Organization Himeji Medical Center, 68, Hommachi, Himeji, Hyogo, 670-8520, Japan; Department of Radiology, National Hospital Organization Himeji Medical Center, 68, Hommachi, Himeji, Hyogo, 670-8520, Japan; Department of Radiology, National Hospital Organization Himeji Medical Center, 68, Hommachi, Himeji, Hyogo, 670-8520, Japan; Department of Radiology, National Hospital Organization Himeji Medical Center, 68, Hommachi, Himeji, Hyogo, 670-8520, Japan; Department of Radiology, National Hospital Organization Himeji Medical Center, 68, Hommachi, Himeji, Hyogo, 670-8520, Japan; Department of Radiology, National Hospital Organization Himeji Medical Center, 68, Hommachi, Himeji, Hyogo, 670-8520, Japan; Department of Therapeutic Radiology, National Hospital Organization Himeji Medical Center, 68, Hommachi, Himeji, Hyogo, 670-8520, Japan; Hiroshima High-Precision Radiotherapy Cancer Center, Hiroshima, 3-2-2, Futabanosato, Higashiku, Hiroshima, 732-0057, Japan; Department of Radiation Oncology, Graduate School of Biomedical and Health Sciences, Hiroshima University, 1-2-3, Kasumi, Minamiku, Hiroshima, 734-8553 Japan; Hiroshima High-Precision Radiotherapy Cancer Center, Hiroshima, 3-2-2, Futabanosato, Higashiku, Hiroshima, 732-0057, Japan; Department of Radiation Oncology, Graduate School of Biomedical and Health Sciences, Hiroshima University, 1-2-3, Kasumi, Minamiku, Hiroshima, 734-8553 Japan

**Keywords:** adaptive radiotherapy (ART), helical tomotherapy (HT), mass density (MD), megavoltage computed tomography (MVCT) number, tolerance levels

## Abstract

Daily dose distributions for adaptive radiotherapy (ART) using helical tomotherapy (HT) are calculated using megavoltage computed tomography (MVCT). Generally, the MVCT number is converted to mass density (MD) using an MD calibration table (MVCT-MD table). The aims of this study are to calculate the tolerance levels of the MD for ART and to evaluate the tolerance levels using clinical patient plans. These tolerance levels of MD were calculated based on the tissue maximum ratio (TMR) of 6MV flattening-filter-free (FFF) beam of HT and the effective tissue thickness data from an International Commission on Radiological Protection 110 phantom data for lung, adipose/muscle and cartilage/spongy-bone. These tolerance levels were determined by considering both the MD causing a dose error of 2% and the variation in MVCT numbers. Subsequently, the stability of the MD values was estimated with the standard deviations (SD) in the MVCT number over 6 months. The dose distribution for clinical patient plans was calculated using the MVCT-MD table with added tolerance levels. These tolerance levels were determined as MD differences causing a dose error of 2%, and were ± 0.049 g/cm^3^, ± 0.030 g/cm^3^ and ± 0.049 g/cm^3^ for lung, adipose/muscle and cartilage/spongy-bone, respectively. The calculated dose distribution errors using the MVCT-MD table added tolerance levels were within 2%. We proposed these tolerance levels in MD for the quality control of the MVCT-MD table.

## INTRODUCTION

Helical tomotherapy (HT) (Accuray Inc., Madison, WI, USA) megavoltage computed tomography (MVCT) images are used for image-guided radiotherapy to improve the precision and accuracy of treatment delivery [1, 2]. MVCT images are also used in adaptive radiotherapy (ART) to evaluate dose distributions and modify treatment plans according to body shape changes and tumor shrinkage during the treatment period [3]. The accuracy of this dose calculation for ART is important for calculating the accurate doses received by the tumor and normal tissues.

The MVCT numbers (Hounsfield unit [HU]) are converted to mass density (MD) [g/cm3] according to a MVCT number to MD calibration (MVCT-MD) table for dose calculation of ART. The accuracy of the MVCT-MD table is critical for dose calculation in inhomogeneous medium. A previous study reported that dose calculation accuracy using MVCT images with the TomoTherapy Hi-ART system (Accuray Inc., Madison, WI, USA) is similar to kilovoltage CT (kVCT) dose calculations [4]. However, several studies have been reported that changes in the MVCT-MD table due to variations in the MVCT number lead to uncertainties in the calculations [5, 6]. Therefore, a quality assurance (QA) method for the stability and reproducibility of the MVCT number has been developed to improve the accuracy of dose calculations for ART [5, 7]. HT guidelines recommend tolerances of ±30 HU, ± 50 HU and ± 50 HU from the baseline for water, lung and bone MVCT numbers, respectively, to achieve a dose calculation accuracy of 2% [7]. The another study calculated the tolerance levels for the ﻿CT number to relative electron density (CT-RED) of water, lung and bone, which has a 2% dose error in linear accelerators (4 MV, 6 MV, 6 MV flattening-filter-free [FFF], 10 MV, 10 MVFFF, 15 MV) and Co-60 from the tissue maximum ratio (TMR) data and effective depth [8, 9]. In these reports, 2–8 beam field 3-dimensional radiotherapy (3DCRT) with general-purpose linear accelerators were evaluated for multiple treatment plans [8, 9]. However, dose distributions for helical intensity-modulated radiation therapy (IMRT) using MVCT images for ART have not been researched. MVCT image-based ART, the standard tool for tomotherapy, is used in the chest and head and neck regions where weight loss and tumor shrinkage often occur [3, 10].

Therefore, this study aimed to propose the MD tolerance levels (*TL*_MD_) for ART. We calculated the *TL*_MD_ that caused a 2% dose error for ART using MVCT images from HT, according to previous work [8, 9]. Furthermore, we confirmed the stability of the daily MVCT number and proposed tolerance levels for the lung, adipose/muscle and cartilage/spongy-bone in the MVCT-MD table. The proposed *TL*_MD_ calculated for ART was validated by adding dose errors to the MVCT-MD table for these clinical patient plans.

## METHODS AND MATERIALS

### MD tolerance levels

#### Difference in MD levels that result in 2% dose error

The depth dose is expressed as a function of the effective depth and the TMR. The relative electron density (RED) error causing the dose error is defined by the following equation (1), corresponding to the equivalent thickness of water [8]:

(1) \begin{equation*} \Delta{\rho}_{\mathrm{e}.i}=\frac{\Delta D/D}{t_i}\frac{TMR}{\left(\frac{dTMR}{d\left({d}_{\mathrm{e}\mathrm{ff}}\right)}\right)} \end{equation*}

Here, *i* denotes the tissue index; Δ*ρ*_e*.i,*_ and *t_i_* denote the RED error and the tissue thickness of index *i*, respectively; *ΔD/D* denotes the relative dose error to the local dose; *TMR* denotes the tissue maximum ratio; *d*_eff_ denotes effective depth; (*dTMR*/*d*(*d*_eff_))/*TMR* is the gradient of TMR relative to the local TMR.

The RED tolerance levels that cause 2% dose error (${TL}_{\mathrm{RED}}^{2\%})$were converted to MD tolerance levels $({TL}_{\mathrm{MD}}^{2\%})$using the conversion coefficient, as seen in equation (2) [11]:

(2) \begin{equation*} {TL}_{\mathrm{MD}}^{2\%}=C\times{TL}_{\mathrm{RED}}^{2\%} \end{equation*}

Here, ${TL}_{\mathrm{MD}}^{2\%}$ and ${TL}_{\mathrm{RED}}^{2\%}$ denote the tolerance levels of MD and RED, respectively; *C* is the conversion factor between MD and RED for human tissues. Conversion factors were 1.009, 1.005 and 1.015 for the lung, adipose/muscle and cartilage/spongy-bone, respectively, based on a previous study [11].

The ${TL}_{\mathrm{MD}}^{2\%}$depends on the energy of the treatment beam and is not influenced by CT scanner. The TMR used for *(dTMR/d(d*_eff*)*_*))/TMR* was acquired using a TomoTherapy HD system (Accuray Inc., Sunnyvale, CA). This system was used to generate the photon beam (6 MV FFF) with an irradiation field of 10 cm × 5 cm. The TMR effective depth gradient at a depth of 10 cm was 3.2% cm^−1^. The TMR data was measured in a water-equivalent phantom (Kyoto Kagaku Co., Kyoto, Japan) from 0 to 25 cm with a source-to-axis distance (SAD) of 85 cm. The recommended accuracy of the calculated dose distribution was less than 2% in heterogeneous materials [12]. Therefore, we applied 2% of *ΔD/D* for relative dose error to the local dose. The effective tissue thicknesses of each tissue type were required to evaluate the dose error with RED in a previous study [8]. The effective tissue thicknesses of each tissue were estimated based on the maximum tissue thickness from an International Commission on Radiological Protection110 (ICRP-110) standard phantom for adults [13]. The effective tissue thicknesses for lung, adipose/muscle and cartilage/spongy-bone tissue were 10, 20 and 10 cm, respectively [9].

#### Variation of the MVCT number

**Fig. 1:**
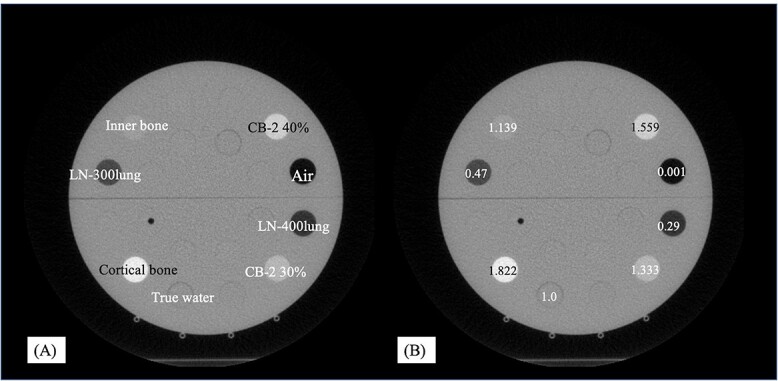
The names (A) and nominal MD values (g/cm^3^) (B) of the tissue equivalent density plugs inserted into the cheese phantom.

To determine the *TL*_MD_, the stability of the MD values was estimated with standard deviations (SD) of MVCT number over six months in our institution. MVCT images of a cheese phantom (Accuray Inc. Madison, WI, USA) with eight tissue equivalent density plugs (Gammex, Sun Nuclear Corporation, USA) were acquired using a HD system. The tissue equivalent density plug used lung to bone with MD values of 0.29–1.822 g/cm^3^. These MVCT images were acquired in normal mode with a slice thickness of 2 mm. These MVCT numbers for air and the water-equivalent substance of cheese phantom were adjusted to −1000 and 25 HU once a week, respectively. The acquisition of the MVCT number for each tissue equivalent density plug (diameter = 20 mm) was measured using ImageJ software (v1.51) on the MVCT images. Data were collected over 6 months (*n* = 78). Figure 1 shows MVCT images of a cheese phantom with eight tissue equivalent density plugs. The root mean square (RMS) of the MVCT number for all tissue-equivalent density plugs was converted to the variation of MD, ${\delta}_{\mathrm{MD}}$. ${\delta}_{\mathrm{MD}}$ is given by equation (3).

(3) \begin{equation*} {\delta}_{\mathrm{MD}}=\sqrt{\frac{1}{n}\sum_{j=1}^n{\delta}_j^2} \end{equation*}

**Table 1:** Treatment planning characteristics: case, prescription, fractions, treatment parameters

Case	Prescription	Fractions	FW (cm)	Pitch	Initial MF	Final MF
Brain	50 Gy to D_90%_ of PTV	20	2.5	0.215	2.000	1.791
Head and neck	70 Gy to D_95%_ of PTV	35	2.5	0.430	2.500	1.941
Chest	70 Gy to D_95%_ of PTV	35	2.5	0.430	2.000	1.891
Prostate	78 Gy to D_95%_ of PTV	39	2.5	0.430	2.000	1.539

PTV, planning target volume; D_x%_, dose received by ≥ x% of volume; FW, field width; MF, modulation factor.

Here, *j* denotes tissue equivalent density plug index; ${\delta}_j$ denotes the variation of MD converted from the SD of MVCT number using MVCT-MD table for each tissue-equivalent density plug; *n* denotes the number of tissue equivalent density plug.

#### Overall tolerance levels of MD

The variation of MVCT number is larger than that of kVCT number [9]. The tolerance levels should include the variation of MVCT number. Therefore, we considered as the variation of the MVCT number using equation (3) and added it to the tolerance levels. Finally, the overall *TL*_MD_ were calculated using the following equation (4):

(4) \begin{equation*} TL_{\mathrm{MD}}=\sqrt{\ {\left({TL}_{\mathrm{MD}}^{2\%}\right)}^2+{\left(2{\delta}_{\mathrm{MD}}\right)}^2} \end{equation*}

### Verification of MD tolerance levels using clinical patient treatment plans

**Fig. 2:**
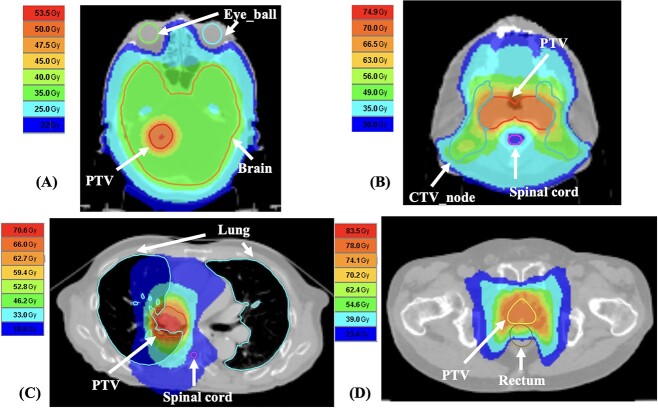
Treatment planning images of (A) the brain, (B) the head and neck, (C) the chest, (D) the Prostate cases. PTV, planning target volume.

Multiple helical IMRT treatment plans based on kVCT images were created using the TomoTherapyHDA™ planning station v5.1.1 (Accuray Inc., Sunnyvale, CA). Table 1 showed the parameters of the treatment plans. The dose distributions were compared with and without the addition of *TL*_MD_ to MVCT-MD table. Dose distributions of treatment planning for brain, head and neck, chest and prostate are shown in Fig. 2. Daily MVCT images acquired with the HD system were used to calculate the ART treatments. This study was approved by the Institutional Review Board (IRB). Dose calculations for the ART treatments and ﻿rigid image registration between the kVCT and MVCT images were performed using planned adaptive software (Accuray Inc., Sunnyvale, CA). The dose calculations were performed using convolution/superposition algorithms. A modified MVCT-MD table was created by adding the *TL*_MD_ to the original table in the radiation therapy planning system (RTPS). *TL*_MD_ is calculated for the effective maximum tissue thicknesses of lung, adipose/muscle and cartilage/spongy-bone from the RED tolerance levels for tissue thickness with a relative dose error of 2% from equation (1) converted to MD from equation (2), and added MVCT number variation equation (4). In previous study, the MD ranges were determined as 0.2 to 0.8 g/cm^3^, 0.9 to 1.07 g/cm^3^ and greater than 1.07 g cm^3^ for lung, adipose/muscle and cartilage/spongy, respectively [13, 14] The dose calculation including the addition of tolerance levels was performed by adding *TL*_MD_ to MVCT-MD table for each tissue equivalent density plug. Hence the MDs from 0.1 to 0.9 g/cm^3^, 1.0 g/cm^3^ and 1.1 to 1.9 g/cm^3^ were added to the *TL*_MD_ for the lung, adipose/muscle and cartilage/spongy-bone regions, respectively. The dose distribution was calculated using the MVCT-MD table with and without the addition of the calculated *TL*_MD_. The MVCT-MD table added the *TL*_MD_, and the calculated plan were defined as ‘modified plans’. Dose-volume histograms were compared between the original and modified plans. The planning target volume (PTV) dosimetric parameters were computed to evaluate the dose assessment metrics D_98%_, D_2%_, homogeneity index (HI) and conformity index (CI) which are recommended in the ICRU report 83 [15]. D_max_, D_mean_, D_10%_ of the organs at risk (OARs) and V_20Gy_ of lung were calculated to evaluate the difference between original and modified plans.

## RESULTS

### Difference in the MD levels that result in 2% dose error

**Fig. 3:**
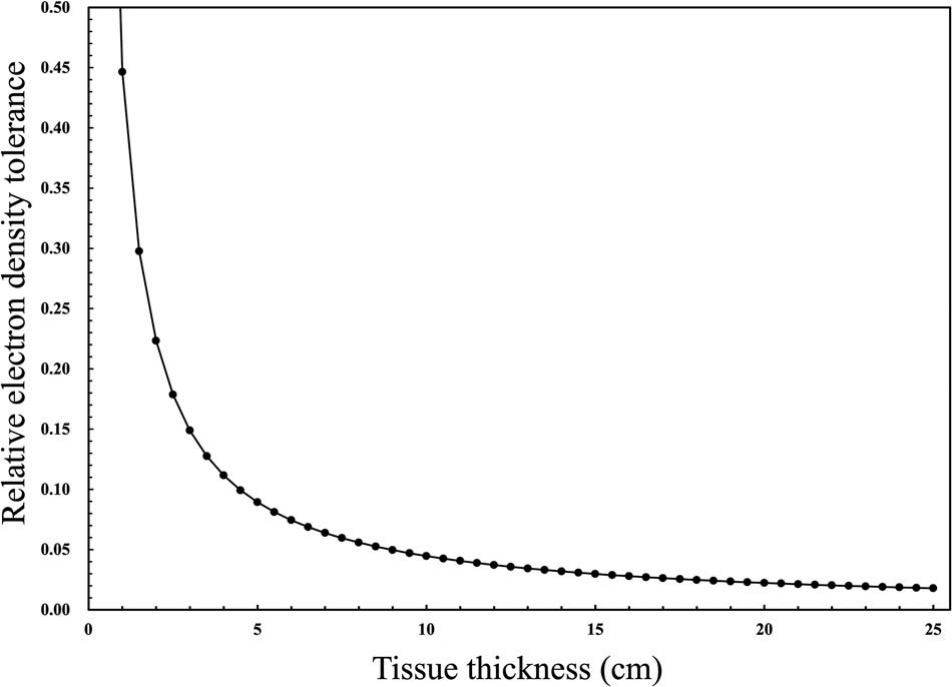
Relationship between the relative RED tolerance for tomotherapy (6 MV FFF) and the tissue thickness corresponding to a dose error of 2%.

Figure 3 displays the relationship between the RED tolerance levels and tissue thickness (cm) corresponding to a dose error of 2% using equation (1). The effective tissue thicknesses of lung, adipose/muscle and cartilage/spongy-bone tissue groups were 10, 20 and 10 cm, respectively [9]. Thus, these tolerance levels of RED corresponding to a dose error of 2% were ± 0.045, ± 0.022 and ± 0.045 for the lung, adipose/muscle and cartilage/spongy-bone, respectively. Additionally, these tolerance levels of RED were converted into ${TL}_{\mathrm{MD}}^{2\%}$ using equation (2). The ${TL}_{\mathrm{MD}}^{2\%}$ were ± 0.045 g/cm^3^, ± 0.022 g/cm^3^ and ± 0.045 g/cm^3^ for the lung, adipose/muscle and cartilage/spongy-bone regions, respectively.

### Stability of the MVCT number

Table 2 shows the variation in MVCT number over six months (*n* = 78) for each material and the converted MD from MVCT number with MVCT-MD table. The ${\delta}_{\mathrm{MD}}$was calculated by equation (3). The RMS of MVCT number for all plugs was 9.2 (HU), which corresponds to the ${\delta}_{\mathrm{MD}}$of 0.010 g/cm3. The ${\delta}_{\mathrm{MD}}$ was converted MVCT number to MD in the MVCT-MD table.

**Table 2:** The MDs, the measured MVCT numbers, and the converted MD from MVCT numbers with MVCT-MD table for tissue equivalent plugs inserted into the cheese phantom

Tissue equivalent density plugs	MD (g/cm^3^)	MVCT number [HU]	Converted MD [g/cm^3^]		
		Mean	SD	Mean	SD
Air	0.001	−936.8	4.3	0.023	0.005
LN-300lung	0.29	−677.0	7.6	0.295	0.008
LN-450lung	0.47	−508.7	9.6	0.480	0.011
True water	1	16.2	8.7	1.057	0.010
Inner bone	1.139	103.7	10.3	1.154	0.011
CB2 30%	1.333	284.3	10.2	1.352	0.011
CB2 50%	1.599	459.5	9.1	1.545	0.010
Cortical bone	1.822	677.4	12.1	1.785	0.013
All plugs RMS			9.2		0.010

MVCT, megavoltage computed tomography; HU, Hounsfield unit; MD, mass density; SD, standard deviation; RMS, root mean square.

### Overall tolerance levels of the MD

**Fig. 4:**
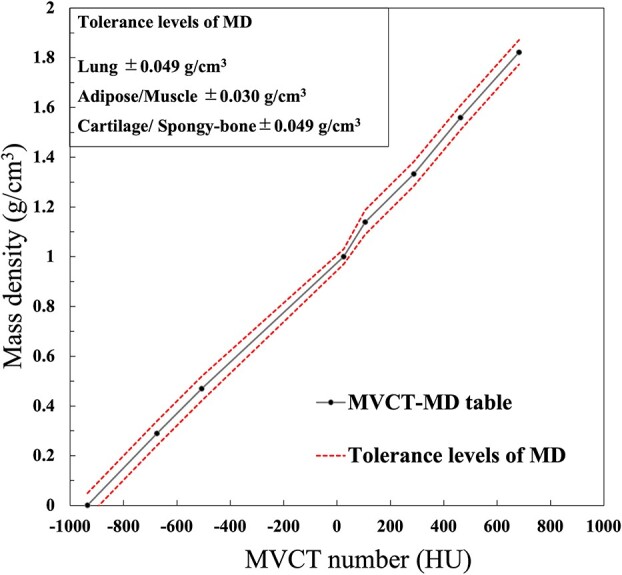
Relationship between MVCT number and MD for the cheese phantom. The black line is from the MVCT-MD table, and the red dashed lines represent the MVCT-MD table data ± the proposed MD tolerance levels.

Table 3 shows the *TL*_MD_ for each MD and tissue group. The *TL*_MD_ were calculated by including the variation of the MVCT number and estimated to cause 2% relative dose error to local dose error at tissue group. The *TL*_MD_ of the MD were ± 0.049 g/cm^3^, ± 0.030 g/cm^3^ and ± 0.049 g/cm^3^ for the lung, adipose/muscle and cartilage/spongy-bone regions, respectively. Figure 4 shows the MVCT -MD table added overall tolerance levels of the MD.

**Table 3:** MD tolerance levels for each tissue group

Tissue group	MD (g/cm^3^)	${TL}_{\mathrm{MD}}^{2\%}$	$2{\delta}_{\mathrm{MD}}$	*TL*_MD_
Lung	0.1–0.9	±0.045	±0.020	±0.049
Adipose/muscle	1.0	±0.022	±0.020	±0.030
Cartilage/spongy bone	1.1–1.9	±0.045	±0.020	±0.049

MD, mass density; ${TL}_{\mathrm{MD}}^{2\%}$, The MD tolerance levels that cause 2% dose error; ${\delta}_{\mathrm{MD}}$, The variation of MD converted from the SD of MVCT number using MVCT-MD table for each tissue-equivalent density plug; *TL*_MD_, overall tolerance levels of MD.

### Validation of MD tolerance using clinical patient treatment plans

Table 4 summarizes the dose assessment metric differences of the brain, head and neck, chest and prostate cases. The dose metric difference between the original and modified plans for the brain and head and neck cases were within −1.7% and −1.6%, respectively. The maximum dose metric difference in the D_2%_ of PTV and D_mean_ of lung of the chest were −1.1% and −1.3%, respectively. The maximum dose metric difference in the D_98%_ of PTV and D_max_ of rectum for the prostate were −2% and −1.9%, respectively, which were equal to or just below the proposed *TL*_MD_ of 2%. The HI result for the brain was 0.01, and CI result for the prostate was 0.02. The HI and CI results did not exhibit any differences.

**Table 4:** The dose assessment metric differences between the original and modified plans for PTV and OAR

Case	Structure name	Metric	Difference	Case	Structure name	Metric	Difference
Brain	PTV	D_98%_	−1.2%	Chest	PTV	D_98%_	−0.9%
		D_2%_	−1.7%			D_2%_	−1.1%
		HI	0.01			HI	0.00
		CI	0.00			CI	0.00
	Eyeball R	D_max_	−1.4%		Lung	D_mean_	−1.3%
	Eyeball L	D_max_	−0.7%			D_10%_	−0.4%
	Brain	D_max_	−1.4%			V_20 Gy_	−0.7%
		D_mean_	−1.1%		Spinal cord	D_max_	−1.0%
Head and neck	PTV	D_98%_	−1.0%	Prostate	PTV	D_98%_	−2.0%
		D_2%_	−0.9%			D_2%_	−1.9%
		HI	0.00			HI	0.00
		CI	0.00			CI	0.02
	CTV node	D_10%_	−1.0%		Rectum	D_max_	−1.9%
	Brainstem	D_max_	−1.6%			D_mean_	−1.2%
	Spinal cord	D_max_	−0.9%			D_10%_	−1.8%

CTV, clinical target volume; PTV, planning target volume; HI, homogeneity index; CI, conformity index; D_x%_, dose received by ≥ x% of volume; V_x Gy_, percentage of volume receiving x Gy of the prescribed dose.

## DISCUSSION

The aim of this study was to propose *TL*_MD_ in ART and evaluate the proposed tolerance levels. The variation in the SD of the MVCT number over six months was 2–8 times larger than that of the kVCT number from a previous study (Table 2) [9]. MVCT images are created with higher energy than kVCT images, which implies that noise increases due to the dominance of Compton interactions. The variation in MVCT numbers should be included in the tolerance levels. The HT guideline recommends that MVCT number tolerances of ±30 HU, ± 50 HU and ± 50 HU be used for water, lung and bone, respectively [7]. When these values were converted to MD using the MVCT-MD table registered in RTPS, they were ± 0.033 g/cm^3^ ± 0.055 g/cm^3^ and ± 0.055 g/cm^3^, respectively [7]. These values are almost consistent with the proposed *TL*_MD_. These factors that determine *TL*_MD_ are ${TL}_{\mathrm{MD}}^{2\%}(\mathrm{equation}\ [1,2])$and ${\delta}_{\mathrm{MD}}$ (equation (4)). ${TL}_{\mathrm{MD}}^{2\%}$depends only on the energy of the treatment beam. We determined this first term by measuring the TMR for the HD system. We did not measure the TMR in the latest model of TomoTherapy (Radixact) and the previous model of TomoTherapy (HI-ART), we expect that it will be close to the ${TL}_{\mathrm{MD}}^{2\%}$for HD system determined in this study. Therefore, the first term (${TL}_{\mathrm{MD}}^{2\%}$) can be used to other facilities, but the second term (δ_MD_) is the variation in MVCT number, which differs significantly among HI-ART, HD system and Radixact and must be evaluated in each model. The SD of MVCT number for HI-ART is 13.0 HU at 9 months [4]. The SD of MVCT number for the HD system was 9.2 HU at 6 months (Table 2). There are no results of stability for Radixact, but reports indicate that the noise is one-third of that of the HD system, so it is expected to be less variable [16]. The difference in SD of MVCT number between HI-ART and HD system is 3.8 HU. This effect increases the *TL*_MD_ of the lung from 0.049 g/cm^3^ to 0.053 g/cm^3^ in HI-ART. This study is the result of a single HD system. To use *TL*_MD_ at other facilities, you need to estimate the MD of the MVCT for each facility.

In the brain, head and neck, chest and prostate clinical treatments performed using HT, the MD values calculated in this study can be used as the proposed *TL*_MD_ for patients. A previous study showed that those generated using the TMR method are dependent on the tissue thickness [8]. In this study, the dose errors in the brain and chest were approximately 1%, while those in the head and neck and prostate cases, were approximately 2%, which is equal to the proposed *TL*_MD_ of 2% (Table 3). For the prostate, as the tissue is thicker than the brain, head and neck and the center of the body, the MD difference added to the MVCT-MD table accumulated along the treatment beam resulted in a dose error close to 2%. The prostate may exhibit a dose difference of more than 2% owing to variations in body thickness. A previous study reported that emphysema decreases lung density to 0.11 g/cm^3^, wherein the normal lung density is approximately 0.25 to 0.37 g/cm^3^ [17]. Assuming the same lung thickness of 20 cm and a density of 0.27 g/cm^3^ and 0.37 g/cm^3^, this lung MD difference would result in a dose error of 0.2%. If we assume a standard human lung from ICRP 110 [13], the dose error may increase.

A previous study reported that tissue equivalent density plugs inserted into the phantom at the CT-RED table differed the physical densities of human tissues, and some tissue equivalents exceeded the density tolerance for a dose error of 2% [17]. The results of this study may differ owing to the difference in the nominal density and actual human tissue of physical density.

## CONCLUSION

In this study, we proposed for *TL*_MD_ the MVCT-MD table for ART dose calculations. The QA of the MVCT-MD table using HT for ART recommends these tolerance values of the MD to be ±0.049 g/cm^3^, ± 0.030 g/cm^3^ and ± 0.049 g/cm^3^ for the lung, adipose/muscle and cartilage/spongy-bone, respectively, indicating a dose error within 2%.

## References

[1] Mackie TR . History of tomotherapy. Phys Med Biol 2006;51:427–53. 10.1088/0031-9155/51/13/R24 16790916

[2] Yartsev S, Kron T, Van Dyk J. Tomotherapy as a tool in image-guided radiation therapy (IGRT): current clinical experience and outcomes. Biomed Imaging Interv J 2007;3:1–8. 10.2349/biij.3.1.e17 PMC3097649 21614258

[3] Ramsey CR, Langen KM, Kupelian PA et al. A technique for adaptive image-guided helical tomotherapy for lung cancer. Int J Radiat Oncol Biol Phys 2006;64:1237–44. 16446055 10.1016/j.ijrobp.2005.11.012

[4] Langen KM, Meeks SL, Poole DO et al. The use of megavoltage CT (MVCT) images for dose recomputations. Phys Med Biol 2005;50:4259–76. 16148392 10.1088/0031-9155/50/18/002

[5] Crop F, Bernard A, Reynaert N. Improving dose calculations on tomotherapy MVCT images Improving dose calculations on tomotherapy MVCT images. J Appl Clin Med Phys 2012;13:241–53. 10.1120/jacmp.v13i6.3986 PMC5718528 23149791

[6] Yadav P, Tolakanahalli R, Rong Y et al. The effect and stability of MVCT images on adaptive TomoTherapy. J Appl Clin Med Phys. 2010;11:4–14. 10.1120/jacmp.v11i4.3229 PMC5720397 21081878

[7] Langen KM, Papanikolaou N, Balog J et al. QA for helical tomotherapy: Report of the AAPM Task Group 148. Med Phys 2010;37:4817–53. 20964201 10.1118/1.3462971

[8] Kilby W, Sage J, Rabett V. Tolerance levels for quality assurance of electron density values generated from CT in radiotherapy treatment planning. Phys Med Biol 2002;47:1485–92. 12043814 10.1088/0031-9155/47/9/304

[9] Nakao M, Ozawa S, Yamada K et al. Tolerance levels of CT number to electron density table for photon beam in radiotherapy treatment planning system. J Appl Clin Med Phys 2018;19:271–5. 10.1002/acm2.12226 PMC5768003 29152898

[10] Castelli J, Simon A, Lafond C et al. Adaptive radiotherapy for head and neck cancer. Acta Oncol (Madr) 2018;57:1284–92. 10.1080/0284186X.2018.1505053 30289291

[11] Nakao M, Ozawa S, Yogo K et al. Tolerance levels of mass density for CT number calibration in photon radiation therapy. J Appl Clin Med Phys 2019;20:45–52. 10.1002/acm2.12601 PMC6560312 31081175

[12] Rock L, Battista JJ, Regional L. et al. Tissue inhomogeneity corrections for megavoltage photon beams. AAPM Report 85, 2004.

[13] International Commission on Radiological Protection . Adult Reference Computational Phantoms, Vol. 39. ICRP Publication 110. Ann. ICRP, 2009. 10.1016/j.icrp.2009.09.001 19897132

[14] Kanematsu N, Inaniwa T, Nakao M. Modeling of body tissues for Monte Carlo simulation of radiotherapy treatments planned with conventional x-ray CT systems. Phys. Med. Biol. 2016;61:5037–50. 10.1088/0031-9155/61/13/5037 27300449

[15] Menzel H-G . International Commission on Radiation Units and Measurements. J ICRU 2015;15:1–2. 10.1093/jicru/ndw028 27335497

[16] Tegtmeier RC, Ferris WS, Bayouth JE *et al.* Characterization of imaging performance of a novel helical kVCT for use in image-guided and adaptive radiotherapy. J Appl Clin Med Phys. 2022;23:e13648. 10.1002/acm2.13648 PMC9194993 35570390

[17] Garnett ES, Webber CE, Coates G. Lung density: clinical method for quantitation of pulmonary congestion and edema. Can Med Assoc J 1977;116:153–4. 608146 PMC1878992

